# Association Between Gastroesophageal Reflux Disease and Frequency of Asthma Exacerbations: A Systematic Review and Meta-Analysis

**DOI:** 10.7759/cureus.105481

**Published:** 2026-03-19

**Authors:** Ruma Akhter, Debopriya Das, Mohammad Mohi Uddin, Sudipta Das, Tamanna Islam Eva, Md Sadiq Uddin Khan, Nahid Khan, Afida Khairul

**Affiliations:** 1 Department of Internal Medicine, Al-Reza General Hospital, Jamalpur, BGD; 2 Department of Nephrology, Sajida Hospital Keraniganj, Dhaka, BGD; 3 Department of Surgery, East West Medical College and Hospital, Dhaka, BGD; 4 Department of Medicine, Maa O Shishu Hospital, Noakhali, BGD; 5 Department of Emergency Medicine, Mahbubur Rahman Memorial Hospital and Nursing Institute, Brahmanbaria, BGD; 6 Department of Emergency Medicine, Dr. Sirajul Islam Medical College and Hospital, Dhaka, BGD; 7 Department of Urology, Sher-E-Bangla Medical College and Hospital, Barishal, BGD

**Keywords:** asthma, disease progression, exacerbation, gastroesophageal reflux, gastroesophageal reflux disease, gerd, meta-analysis, systematic review

## Abstract

Gastroesophageal reflux disease (GERD) is common in patients with asthma; however, its role in asthma exacerbations remains unclear due to inconsistent study findings. This systematic review and meta-analysis aimed to synthesize the available evidence on the relationship between GERD and asthma exacerbations through two complementary objectives: (1) to assess the association between the presence of GERD and the frequency of asthma exacerbation in observational studies, and (2) to evaluate the effect of anti-reflux therapy (primarily proton pump inhibitors) on asthma exacerbations in randomized controlled trials (RCTs). Five research databases were searched up to December 2025 for RCTs and observational studies. A total of 10 studies were included, comprising six RCTs and four observational studies. Risk of bias was assessed using Risk of Bias 2 (ROB 2) and Risk of Bias in Non-randomized Studies of Interventions (ROBINS-I) tools. A random-effects model was used to pool the data, and heterogeneity was explored through subgroup analyses. The overall pooled effect was not statistically significant (standardized mean difference (SMD) = −0.12, 95% confidence interval (CI): −0.41 to 0.16, p = 0.323), with high heterogeneity (I² = 77.87%). Subgroup analysis revealed a statistically significant difference based on symptom status. Anti-reflux therapy significantly reduced asthma exacerbations in patients with symptomatic GERD (SMD = −0.41, 95% CI: −0.72 to −0.10), whereas no significant benefit was observed in patients without GERD symptoms. No significant differences were found across subgroups based on study design or diagnostic method. Overall, no significant association between GERD and asthma exacerbations was identified. However, symptom status appeared to act as a clinically significant effect modifier. Anti-reflux therapy may benefit asthmatic patients with symptomatic GERD, but empirical treatment of all asthmatic patients with evidence of reflux is not supported. These findings favor a symptom-driven treatment approach rather than routine empirical therapy.

## Introduction and background

Gastroesophageal reflux disease (GERD) and asthma are two common chronic conditions that frequently coexist, creating a complex clinical interaction with important implications for disease management and patient outcomes. GERD is defined as the retrograde movement of gastric contents into the esophagus and affects approximately 20-40% of adults in Western countries. Asthma, a chronic inflammatory airway disease, affects more than 300 million individuals worldwide [1,2]. Epidemiological studies indicate a significant association between the two conditions, with GERD symptoms reported in up to 60-80% of patients with asthma, a prevalence substantially higher than that of the general population [3].

The pathophysiological relationship between GERD and asthma is considered bidirectional. Micro-aspiration of gastric contents into the tracheobronchial tree may directly induce airway inflammation, bronchoconstriction, and hyperresponsiveness. Additionally, esophageal acid exposure can stimulate chemoreceptors and trigger vagally mediated reflex bronchoconstriction [4]. Conversely, mechanical changes in intrathoracic pressure during asthma attacks, along with the bronchodilatory effects of certain medications such as theophylline, may reduce lower esophageal sphincter pressure, thereby precipitating or worsening reflux [5].

Despite these proposed mechanisms, the clinical impact of GERD on asthma severity, particularly on exacerbation frequency, remains controversial. Previous systematic reviews have yielded conflicting conclusions: some suggest a benefit of proton pump inhibitor (PPI) therapy in selected populations [3], while others find no overall effect, with the inconsistency attributed to heterogeneity in patient selection, GERD definitions, and outcome measures. Asthma exacerbations, defined as acute episodes of progressive worsening of symptoms such as breathlessness, wheezing, and cough, are associated with increased morbidity, reduced quality of life, greater healthcare utilization, and mortality [6]. If GERD acts as a trigger or effect modifier, its effective management could represent an essential component of asthma care. However, clinical trials evaluating PPIs have not consistently demonstrated improvement in asthma control, underscoring the need to clarify this association through rigorous synthesis that accounts for potential effect modifiers [7].

A systematic evaluation of the available evidence is therefore warranted to determine whether there is a measurable relationship between GERD and asthma exacerbations. A comprehensive understanding of the GERD-asthma relationship requires synthesis of both interventional and observational evidence. Observational studies address whether the presence of GERD constitutes a risk factor for exacerbations, informing risk stratification and prognosis. Randomized controlled trials (RCTs) address whether treating GERD modifies exacerbation risk, informing therapeutic decisions. While these study designs answer distinct clinical questions, their combined synthesis allows examination of consistency across evidence types and exploration of whether treatment effects align with epidemiological associations.

This systematic review and meta-analysis was conducted according to the following PICO (population, intervention/exposure, comparison, and outcome) framework: population: patients with physician-diagnosed asthma of any age; intervention/exposure: (a) presence of GERD (diagnosed clinically or objectively) in observational studies, and (b) anti-reflux therapy (primarily PPIs) in RCTs; comparison: asthmatic patients without GERD (observational studies) or placebo/no treatment (RCTs); outcome: frequency of asthma exacerbations, defined as events requiring systemic corticosteroids, emergency department visits, or hospitalization.

The primary objectives were (1) to determine whether the presence of GERD is associated with increased asthma exacerbation frequency in observational studies, and (2) to evaluate whether anti-reflux therapy reduces asthma exacerbation frequency in RCTs. It was hypothesized that both GERD presence and treatment responsiveness would be modified by patient symptom status.

## Review

Methodology

The systematic review and meta-analysis were conducted in accordance with the Preferred Reporting Items for Systematic Reviews and Meta-Analyses (PRISMA) guidelines [8]. The study protocol was not prospectively registered in the International Prospective Register of Systematic Reviews (PROSPERO).

Development and execution of the electronic search strategy

The search strategy was developed in consultation with an experienced medical librarian. The core concepts of the review, gastroesophageal reflux, asthma, and exacerbation, were identified and translated into a comprehensive combination of Medical Subject Headings (MeSH) and free-text keywords. Boolean operators (AND, OR) and truncation were used to capture variations in terminology.

To reduce publication bias, no date restrictions were applied. The search was limited to human studies and articles published in English. The search strategy was adapted to the indexing terms and syntax requirements of each database. The following databases were searched: PubMed, Embase, Cochrane Central Register of Controlled Trials, Scopus, and Web of Science Core Collection. No grey literature sources (such as clinicaltrials.gov or conference abstracts) were searched, and the search was strictly limited to the five listed databases (Table 1).

**Table 1:** Comprehensive search strategy for electronic databases. Abbreviations used in the literature search strategy: MeSH: Medical Subject Headings; TS: topic search; TITLE-ABS-KEY: title, abstract, and keywords; /exp: explode (include all narrower terms in database indexing); GERD: gastroesophageal reflux disease.

Database	Search query components	Applied filters	Syntax/Modifiers
PubMed	("Gastroesophageal Reflux"[Mesh] OR "GERD" OR "gastro-oesophageal reflux" OR "heartburn") AND ("Asthma"[Mesh] OR "asthma" OR "bronchial hyperreactivity") AND ("Disease Progression"[Mesh] OR "Exacerbation"[Mesh] OR "exacerbate" OR "flare-up" OR "attack*" OR "hospitalization" OR "emergency room visit")	Humans; English	Boolean operators (AND, OR); Truncation (*); Field tags [Mesh]
Embase (via Ovid)	('gastroesophageal reflux'/exp OR 'gerd' OR 'gastro-oesophageal reflux') AND ('asthma'/exp OR 'asthma') AND ('disease exacerbation'/exp OR 'disease progression'/exp OR 'exacerbation*' OR 'asthma attack')	Human; English language	Boolean operators; Truncation; Explode (/exp)
Cochrane Central	(gastroesophageal reflux OR GERD) AND asthma AND (exacerbation* OR progression OR hospitalization)	Trials	Boolean operators; Truncation
Scopus	TITLE-ABS-KEY ( ( "gastroesophageal reflux" OR GERD ) AND ( asthma ) AND ( exacerb* OR hospitali* OR "emergency" ) )	Article; Review; English	Boolean operators; Truncation; Field codes (TITLE-ABS-KEY)
Web of Science Core Collection	TS=((gastroesophageal reflux OR GERD) AND asthma AND (exacerbat* OR progression OR hospitalization))	Articles; English	Boolean operators; Truncation; Field tag (TS=Topic)

To make the search as comprehensive as possible and reduce the risk of overlooking relevant studies, a manual search of the reference lists of all included articles and other relevant published systematic reviews was conducted. This additional search aimed to identify essential studies not captured by the electronic database searches.

To ensure methodological rigor and minimize reviewer bias, two reviewers independently performed all stages of the screening process (title/abstract and full-text). Study inclusion or exclusion at each stage was recorded. Disagreements were resolved through discussion between the two reviewers. If consensus could not be reached, a third senior reviewer made the final decision.

Study selection protocol

The inclusion criteria for this meta-analysis were defined according to the PICO framework [9]. The target population included individuals with a diagnosis of asthma. The exposure of interest was a confirmed diagnosis of GERD based on accepted clinical or investigational methods. In studies evaluating treatment, medical therapy for GERD was considered the intervention. The comparator group consisted of asthmatic patients without GERD or, in interventional studies, a placebo control. The primary outcome was the frequency of asthma exacerbations, defined as objectively measured events, including the number of events, incidence rates, or the proportion of patients experiencing one or more exacerbations. An exacerbation was defined as an event requiring increased healthcare utilization or escalation of treatment (Table 2).

**Table 2:** Study eligibility criteria based on the PICO framework. Abbreviations used in the study framework: PICO: population, intervention/exposure, comparison, outcome; GERD: gastroesophageal reflux disease; PPIs: proton pump inhibitors. The PICO framework was applied in this study as described by Scells et al. [9].

PICO element	Inclusion criteria	Exclusion criteria
Population (P)	Human patients of any age or gender with a physician diagnosis of asthma.	Studies on animals, or studies where the cohort does not have confirmed asthma (e.g., chronic cough only).
Exposure/Intervention (I)	Diagnosis of GERD (based on clinical symptoms, validated questionnaire, pH monitoring, or endoscopy). In intervention studies: anti-reflux treatment (e.g., PPI therapy).	Studies where GERD status is not defined or assessed.
Comparison (C)	Asthmatic patients without GERD, or patients receiving a placebo in intervention studies.	Studies without a clear comparator group.
Outcome (O)	Frequency of asthma exacerbations, measured as rate (e.g., events per person-year), count, or proportion of patients experiencing ≥1 exacerbation. Exacerbation is defined as requiring systemic corticosteroids, an emergency department visit, or hospitalization.	Studies not reporting exacerbations as an outcome (e.g., only lung function, symptom scores).
Study design (S)	Observational studies (cohort, case-control, cross-sectional) and randomized controlled trials (RCTs).	Case reports, editorials, commentaries, conference abstracts without full data, and non-English publications.

Systematic data extraction and management

After the final selection of studies, two reviewers independently extracted data using a pre-piloted standardized electronic form designed in Microsoft Excel (Microsoft Corporation, Redmond, WA). The form included data in the following areas: (1) study characteristics: first author, year of publication, country, study design, sample size, and follow-up period; (2) participant characteristics: age, gender, baseline asthma, and GERD criteria; (3) exposure/intervention factors: method of GERD diagnosis or type and dosage of anti-reflux treatment; (4) outcome data: definition of asthma exacerbation, event counts, rates, proportions, odds ratios (OR), and hazard ratios (HR).

Any discrepancies between the two reviewers were resolved by jointly re-examining the source publication. If necessary, a third reviewer adjudicated.

Assessment of risk of bias and publication bias

The methodological quality and risk of bias of each study were assessed using established tools. RCTs were evaluated using the revised Cochrane Risk of Bias 2 (ROB 2) tool [10]. Non-randomized observational studies were assessed using the Risk of Bias in Non-randomized Studies of Interventions (ROBINS-I) tool [11]. Studies were classified as low risk, some concerns, or high risk of bias.

Publication bias was assessed using multiple approaches. Visual inspection of funnel plot asymmetry was complemented by Egger's linear regression test for small-study effects [12], with a p-value < 0.10 suggesting potential bias. Additionally, to adjust for potential publication bias, Duval and Tweedie's trim-and-fill method was applied, which imputes hypothetical missing studies to achieve funnel plot symmetry and recalculates the pooled effect size incorporating these imputed studies.

Quantitative synthesis and heterogeneity investigation

Review Manager (Desktop Version 5.4, 2020, The Cochrane Collaboration, London, UK) [13] was used to conduct all statistical analyses. As the included studies were expected to be heterogeneous in clinical and methodological characteristics, meta-analyses were performed using a random-effects model with the DerSimonian and Laird approach to provide a conservative pooled estimate.

For meta-analysis, all outcome measures were converted to a common metric, i.e., the standardized mean difference (SMD) with 95% confidence intervals. The conversion followed established methodologies. For dichotomous outcomes (event counts, proportions of patients with ≥1 exacerbation), odds ratios were calculated and transformed to SMD using the Hasselblad method: SMD = ln(OR) × (√3/π), with variance derived accordingly. For rate data (events per person-year), incidence rate ratios (IRRs) were converted to log rate ratios, then to SMD using the Cox transformation. For continuous outcomes (symptom scores, lung function), reported means and standard deviations were used directly to calculate SMD (Hedges' g with small-sample correction). For studies reporting multiple exacerbation outcomes, the most clinically relevant definition (e.g., severe exacerbations requiring systemic corticosteroids over mild exacerbations) was prioritized.

Sensitivity analyses were performed using only studies reporting directly compatible outcomes to validate the conversion approach. For dichotomous outcome data (e.g., the proportion of patients with ≥1 exacerbation), pooled odds ratios (ORs) with 95% confidence intervals (CIs) were calculated. IRRs were pooled for rate data. Statistical heterogeneity was assessed using the I² statistic, where values of 25%, 50%, and 75% were considered low, moderate, and high heterogeneity, respectively.

Pre-specified subgroup analyses were performed to investigate sources of heterogeneity, based on GERD diagnostic approach (objective vs. symptomatic), study design (RCTs vs. observational), and GERD symptom status. Sensitivity analyses were conducted by successively removing studies deemed high risk of bias to assess the robustness of the pooled results. All p-values were two-sided, and values < 0.05 were considered statistically significant.

Results

Study Selection Process

**Figure 1:**
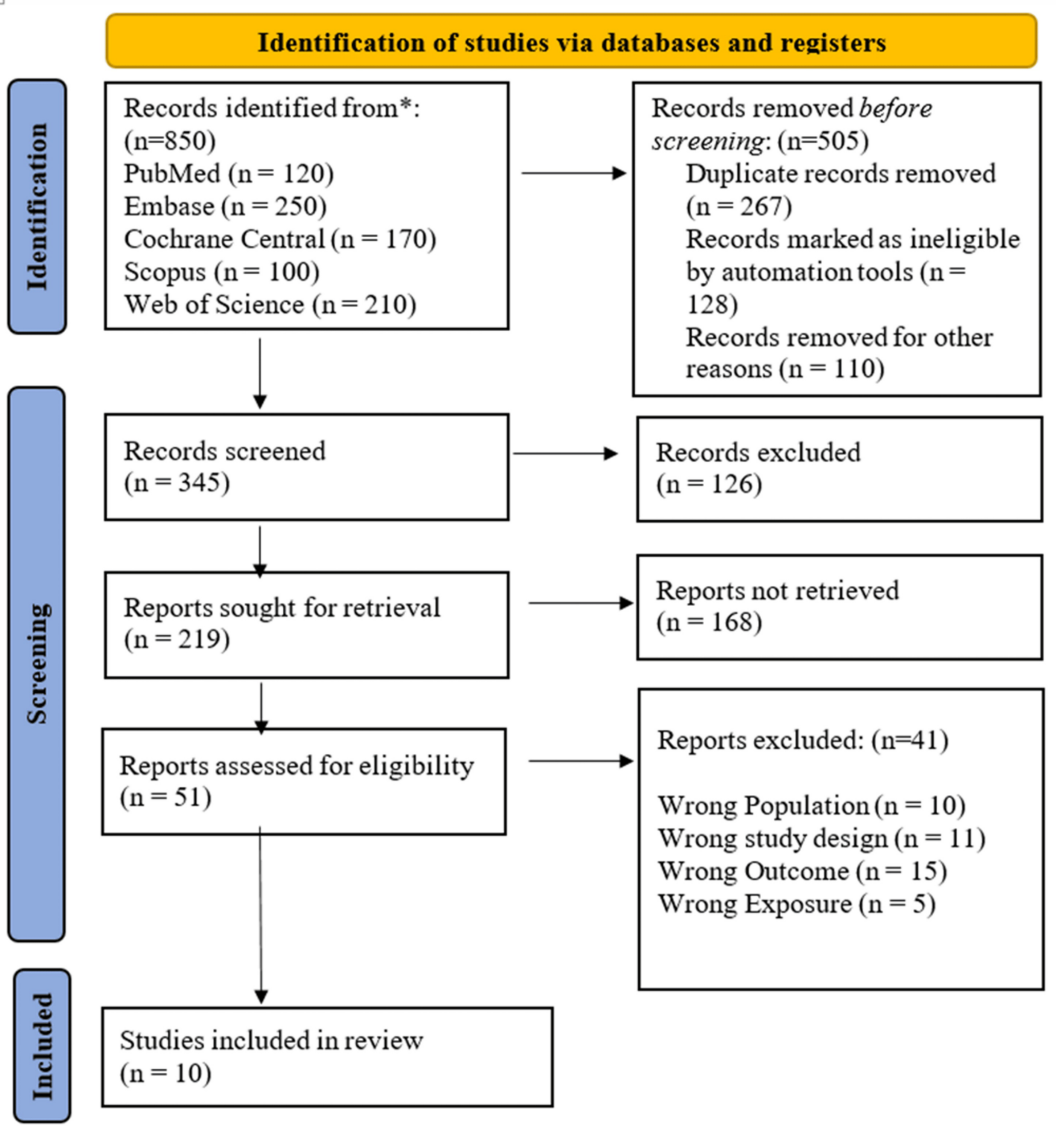
Identification and selection of studies for systematic review. The Preferred Reporting Items for Systematic Reviews and Meta-Analyses (PRISMA) flowchart was applied in this study as described by Page et al. [8].

The initial search was conducted across five databases (PubMed, Scopus, Web of Science, Embase, and Cochrane Central), yielding 850 records. After removal of duplicates and records excluded by automated filters (n = 505), 345 unique records remained for title and abstract screening. This step resulted in the exclusion of 126 records. The remaining 219 reports were assessed at the full-text level; 168 were unavailable, and 51 studies underwent detailed eligibility assessment. Of these, 41 did not meet the predefined PICO criteria. The most frequent reasons for exclusion were an irrelevant primary outcome (n = 15), inappropriate study design (n = 11), and ineligible patient population (n = 10). Ultimately, 10 studies met all eligibility criteria and were included in the final systematic review and meta-analysis (Figure 1) [14-23].

Table 3 shows that the included articles comprised six RCTs and four observational studies conducted in various countries, including the United States of America (USA), multinational cohorts, China, India, and Japan. The study populations mainly consisted of adults with moderate or severe asthma, and GERD was identified based on symptoms, questionnaires, pH monitoring, or endoscopy. The interventions in the RCTs included PPIs (esomeprazole, lansoprazole, omeprazole, and rabeprazole) compared with placebo. Asthma exacerbation outcomes were reported and measured differently across studies, ranging from investigator-identified worsening to the use of specific scales, such as the Episodes of Poor Asthma Control (EPAC) or severe exacerbation criteria.

**Table 3:** Characteristics and key outcomes of studies included in the meta-analysis on gastroesophageal reflux disease and asthma exacerbations. References correspond to Kiljander et al. [14], Littner et al. [15], DiMango et al. [16], Kiljander et al. [17], Liang et al. [18], Harding et al. [19], Meier et al. [20], Gopal et al. [21], Tsugeno et al. [22], and Shimizu et al. [23]. Abbreviations used: ACT: asthma control test; ACQ: asthma control questionnaire; ATS: American Thoracic Society; BID: twice per day; CI: confidence interval; EGD: esophagogastroduodenoscopy; EPAC: episode of poor asthma control; FEV1: forced expiratory volume in one second; FVC: forced vital capacity; GERD: gastroesophageal reflux disease; GINA: Global Initiative for Asthma; HR: hazard ratio; ICS: inhaled corticosteroids; IRR: incidence rate ratio; LABA: long-acting beta-agonist; LA: Los Angeles classification for esophagitis; NOC: nocturnal asthma; OR: odds ratio; PEF: peak expiratory flow; QUEST: questionnaire for the diagnosis of reflux disease; RCT: randomized controlled trial; RDQ: reflux diagnostic questionnaire; RoB: risk of bias; vs.: versus; yrs: years.

Study author, year, country	Study characteristics	Participant characteristics	Exposure/intervention details	Outcome
Kiljander et al. (2006), multinational [14]	Design: Randomized, double-blind, placebo-controlled, parallel-group. N: 770 randomized. Duration: 16 weeks.	Age: Mean ~45 yrs. Gender: ~72% female. Asthma: Moderate-to-severe, on ICS. Required reversibility. GERD: Stratified by symptoms & nocturnal asthma (NOC). GERD+/NOC+ (n=350).	Diagnosis: Symptom-based (heartburn/regurgitation ≥2/wk) or history of abnormal pH/esophagitis. Intervention: Esomeprazole 40 mg twice daily vs. placebo.	Exacerbation definition: Not explicitly defined; "asthma exacerbation" counted. Events: 22 (esomeprazole) vs. 24 (placebo). Time to exacerbation: Median 42 vs. 67 days (p=0.70).
Littner et al. (2005), USA [15]	Design: Multicenter, randomized, double-blind, placebo-controlled. N: 207 randomized. Duration: 24 weeks.	Age: Mean ~46 yrs. Gender: 67% female. Asthma: Moderate-to-severe persistent, on ICS. FEV1 50-85% predicted. GERD: Symptomatic acid reflux (heartburn/regurgitation).	Diagnosis: Clinician-assessed symptoms (pH monitoring optional). Intervention: Lansoprazole 30 mg twice daily vs. placebo.	Exacerbation definition: Investigator-determined episodic worsening of asthma symptoms. Events (any): 8 pts (12 events) vs. 22 pts (27 events). 8.1% vs. 20.4%; OR 2.9 (95% CI 1.2–6.9), p=0.017. Events (Mod-Sev): 4 pts (8 events) vs. 15 pts (19 events). 4% vs. 13.9%; OR 3.8 (95% CI 1.2-12.0), p=0.016.
DiMango et al. (2009), USA [16]	Design: Randomized, double-blind, placebo-controlled, parallel-group. N: 402 randomized. Duration: 24 weeks.	Age: Mean ~42 yrs. Gender: ~68% female. Asthma: Poorly controlled despite ICS. FEV1 >50% predicted. GERD: Minimal or no symptoms (excluded if heartburn ≥2/wk).	Diagnosis: Ambulatory pH monitoring in all. 40% had asymptomatic pathologic GERD. Intervention: Esomeprazole 40 mg twice daily vs. placebo.	Exacerbation definition: Episode of Poor Asthma Control (EPAC): 30% drop in PEF x2 days, urgent care visit, or oral steroids. Event rate (EPACs/person-yr): 2.5 vs. 2.3. Incidence rate ratio (IRR) 1.1 (95% CI 0.8–1.5), p=0.66.
Kiljander et al. (2010), multinational [17]	Design: Randomized, double-blind, placebo-controlled, parallel-group. N: 961 randomized. Duration: 26 weeks.	Age: Mean ~45 yrs. Gender: ~77% female. Asthma: Moderate-to-severe, on ICS+LABA. Required exacerbation history. GERD: Symptomatic (heartburn/regurgitation ≥moderate severity).	Diagnosis: Symptom-based (Reflux Disease Questionnaire) or history of abnormal pH. Intervention: Esomeprazole 40 mg once daily OR twice daily vs. placebo.	Exacerbation definition: "Severe asthma exacerbation." Events (Placebo/40 mg once a day/40 mg BID): 34 (10%)/32 (10%)/24 (7.5%).
Liang et al. (2013), China [18]	Design: Cross-sectional observational study. N: 768 asthma patients. Duration: Single assessment.	Age: Mean ~46 yrs. Gender: ~60% female. Asthma: Diagnosed per GINA. GERD: 27.6% diagnosed via RDQ (score ≥12).	Diagnosis: Reflux Diagnostic Questionnaire (RDQ). Intervention: None.	Exacerbation definition: Not well-controlled asthma (ACT score <20). Events: GERD associated with not well-controlled asthma: OR 3.12 (95% CI 1.53-4.88) after adjustment.
Harding et al. (1996), USA [19]	Design: Prospective cohort (non-placebo-controlled). N: 30 completed. Duration: Pre-therapy phase (4 weeks) + Acid titration + 3 months of suppressive therapy.	Age: Mean 46 yrs (range 23-70) Gender: 60% female. Asthma criteria: Adult asthmatics (ATS criteria), nonsmokers, 77% moderate-to-severe. GERD criteria: Symptoms (heartburn/regurgitation ≥2/month) + abnormal 24-hr esophageal pH test.	GERD diagnosis: Symptomatic + abnormal 24-hr pH monitoring. Anti-reflux treatment: Omeprazole, dose titrated via pH monitoring. Mean effective dose: 27 mg/day (27% required >20 mg/d).	Exacerbation/response definition: A priori asthma response: >20% reduction in asthma symptom score and/or >20% improvement in PEF. Event counts: 22/30 (73%) were asthma symptom and/or PEF responders. Clinical change: Responders: -57% asthma symptoms (p<0.001), +8-9% PEF (p<0.005).
Meier et al. (1994), USA [20]	Design: Double-blind, placebo-controlled, crossover. N: 15 completed. Duration: Two 6-week treatment periods separated by a 2-week washout.	Age: Mean 49 yrs. Gender: 60% male, 40% female. Asthma criteria: ATS-defined asthma with objective reversibility (>15% FEV1/FVC change). GERD criteria: Endoscopic esophagitis and/or abnormal distal 24-hr pH score (≥22).	GERD diagnosis: EGD (esophagitis) and/or 24-hr pH monitoring (distal score ≥22). Anti-reflux treatment: Omeprazole 40 mg/day (20 mg BID) vs. placebo.	Exacerbation/response definition: ≥20% net improvement in FEV1 on omeprazole vs. placebo (Omeprazole-Responsive Asthma - ORA). Event counts: 4/15 (27%) met ORA criteria. The ORA group had complete endoscopic healing (4/4 vs. 6/11 non-ORA, p<0.01).
Gopal et al., 2005, India [21]	Design: Non-randomized, open-label. Sample: 70 screened → 40 with GERD. Follow-up: 4 weeks	Age: 16–40 years. Gender: 22M, 18F. Asthma: Perennial asthma. GERD: Questionnaire + Bernstein test + fall in FEV₁ > 20%.	GERD diagnosis: Questionnaire (Field criteria) + Bernstein test + spirometry. Intervention: Omeprazole 20 mg/day added to asthma therapy.	Outcome: Symptom score, drug score, spirometry (FVC, FEV₁, PEF). Results: Significant improvement in all scores and spirometry (p < 0.001). No OR/HR provided.
Tsugeno et al., 2003, Japan [22]	Design: Prospective, open-label. Sample: 53 asthmatics (22 GERD, 28 non-GERD). Follow-up: 8 weeks	Age: ~67 years mean. Gender: 20M, 33F. Asthma: ATS criteria. GERD: QUEST score ≥4 or endoscopy findings (LA classification).	GERD diagnosis: QUEST + endoscopy. Intervention: Rabeprazole 20 mg/day	Outcome: PEF improvement ≥20%. Results: 8/21 GERD patients responded. OR for response: QUEST score OR = 1.47 (1.06–2.04); steroid-dependent asthma OR = 0.01 (0.0004–0.31).
Shimizu et al., 2006, Japan [23]	Design: RCT. Sample: 30 asthmatics with GERD. Follow-up: 2 months	Age: ~56–59 years mean. Gender: 13M, 17F. Asthma: ATS criteria. GERD: QUEST ≥4 and/or endoscopy (LA classification with Grade M/N).	GERD diagnosis: QUEST + endoscopy. Intervention: Group 1: Roxatidine 150 mg/day. Group 2: Lansoprazole 30 mg/day.	Outcome: PEF, ACQ score, FEV₁. Results: Lansoprazole improved PEF (p = 0.002) and ACQ (p < 0.05) vs. baseline. Roxatidine did not.

Comparative Risk of Bias Assessment

**Figure 2:**
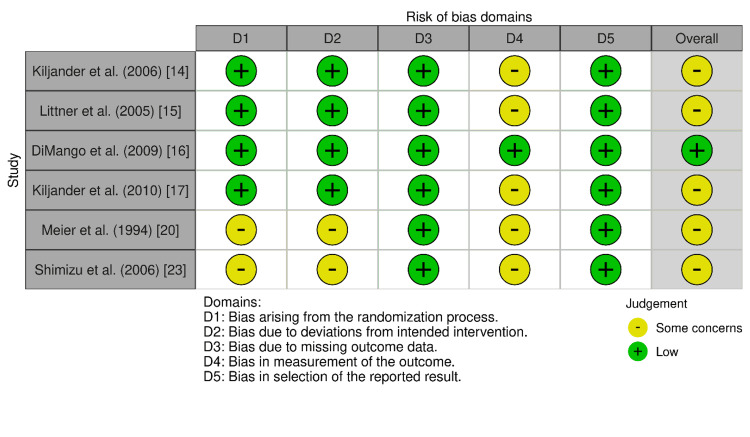
Risk of bias assessment for randomized controlled trials (RoB 2). RoB 2, ROBINS-I, ROBINS-E, and ROB ME are licensed under the Creative Commons Attribution-NonCommercial-NoDerivatives 4.0 International License. Risk of bias was applied in this study as described by Igelström et al. [10]. References correspond to Kiljander et al. [14], Littner et al. [15], DiMango et al. [16], Kiljander et al. [17], Meier et al. [20], and Shimizu et al. [23]. RoB 2: Risk of Bias 2; ROBINS-I: Risk of Bias in Non-randomized Studies of Interventions; ROBINS-E: Risk of Bias in Non-randomized Studies - of Exposures; ROB ME: Risk of Bias due to missing evidence.

When the Cochrane RoB 2 tool was applied to the six RCTs, as shown in Figure 2, one RCT [16] was found to have a low risk of bias across all evaluated domains.

**Figure 3:**
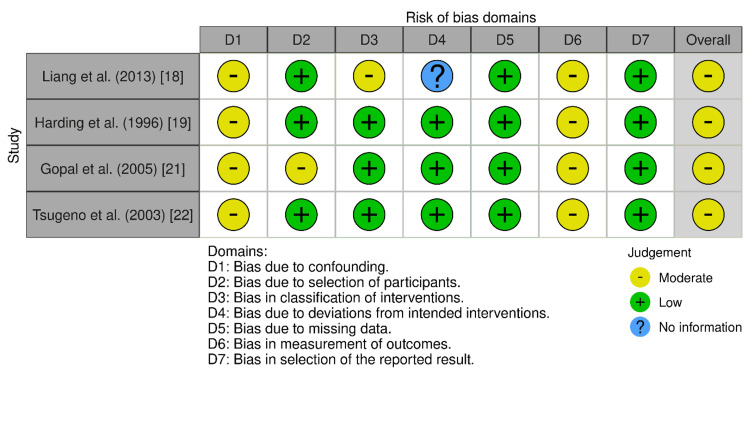
Risk of bias assessment for non-randomized studies (ROBINS-I) RoB 2, ROBINS-I, ROBINS-E, and ROB ME are licensed under the Creative Commons Attribution-NonCommercial-NoDerivatives 4.0 International License. References correspond to Liang et al. [18], Harding et al. [19], Gopal et al. [21], and Tsugeno et al. [22]. Risk of bias was applied in this study as described by Igelström et al. [10]. RoB 2: Risk of Bias 2; ROBINS-I: Risk of Bias in Non-randomized Studies of Interventions; ROBINS-E: Risk of Bias in Non-randomized Studies - of Exposures; ROB ME: Risk of Bias due to missing evidence.

The four non-randomized observational studies, assessed using the ROBINS-I tool (Figure 3), consistently demonstrated a moderate risk of bias across the seven domains.

Publication Bias

**Figure 4:**
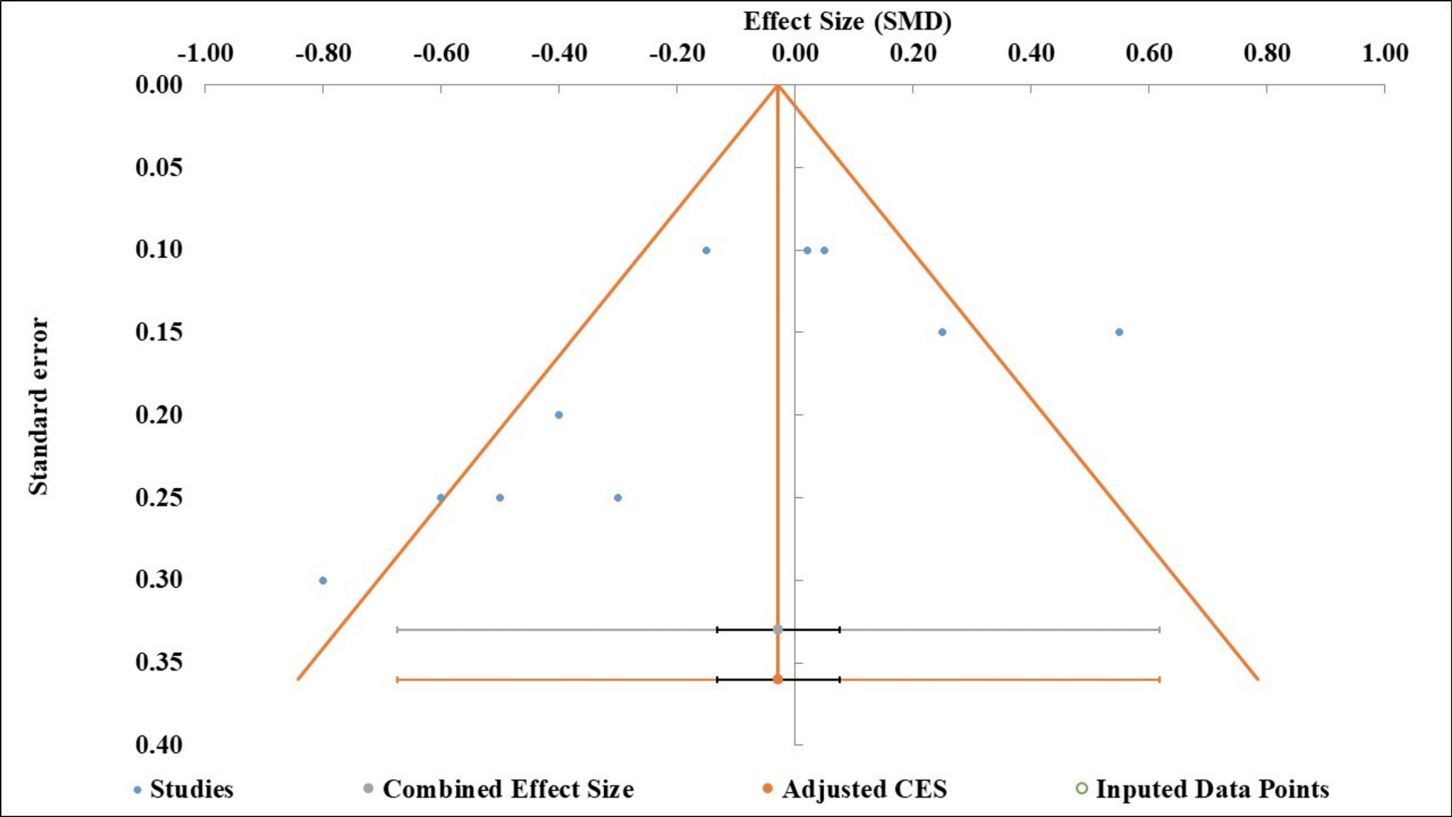
Funnel plot assessing publication bias in the meta-analysis of GERD and asthma exacerbations. The funnel plot was applied in this study as described by Hayashino et al. [12]. GERD: gastroesophageal reflux disease; SMD: standardized mean difference; CES: combined effect size.

The funnel plot showed visual asymmetry, with the majority of studies reporting beneficial effects of GERD treatment, while a few studies appeared on the opposite side, indicating potential publication bias (Figure 4). However, Egger’s regression test for small-study effects was not statistically significant (intercept = -2.72, p = 0.133) (Table 4). This indicates that, quantitatively, there was no substantial evidence of publication bias, although the test may be underpowered, and the visual asymmetry warrants caution when interpreting the pooled results [14].

**Table 4:** Egger's linear regression test for small-study effects. Egger’s test was applied in this study as described by Hayashino et al. [12]. No significant result was observed, using a threshold of p < 0.05. CI represents confidence interval.

Parameter	Estimate	Standard error	95% CI, Lower limit	95% CI, Upper limit
Intercept	-2.72	1.62	-6.39	0.95
Slope	0.34	0.24	-0.20	0.87
t-value	-1.67			
p-value	0.133			

Meta-analysis findings

*Forest* *Plot* *and* *Heterogeneity* *Assessment*

**Figure 5:**
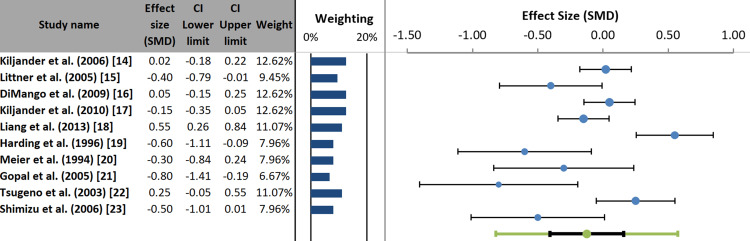
Forest plot of individual and pooled effect sizes for included studies. References correspond to Kiljander et al. [14], Littner et al. [15], DiMango et al. [16], Kiljander et al. [17], Liang et al. [18], Harding et al. [19], Meier et al. [20], Gopal et al. [21], Tsugeno et al. [22], and Shimizu et al. [23]. SMD: standardized mean difference.

The pooled effect size of the 10 studies in the random-effects meta-analysis was -0.12 SMD, which was not statistically significant (95% CI: -0.41 to 0.16; p = 0.323). This indicates no overall effect of GERD treatment or the presence of GERD on the rate of asthma exacerbations. The analysis revealed substantial heterogeneity among studies (I² = 77.87%, p = 0.001) (Figure 5) [24].

*Subgroup* *Analysis*

**Figure 6:**
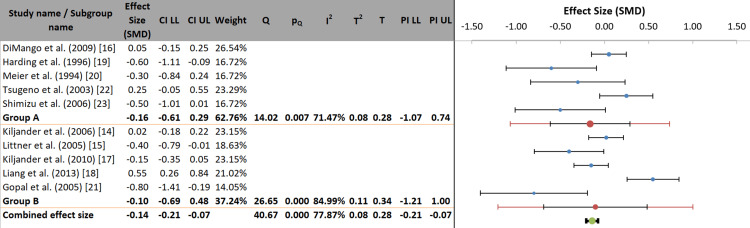
Forest plot of subgroup analysis by the method of GERD diagnosis. References correspond to Kiljander et al. [14], Littner et al. [15], DiMango et al. [16], Kiljander et al. [17], Liang et al. [18], Harding et al. [19], Meier et al. [20], Gopal et al. [21], Tsugeno et al. [22], and Shimizu et al. [23]. GERD: gastroesophageal reflux disease; SMD: standardized mean difference; CI: confidence interval; LL: lower limit; UL: upper limit; PI: prediction interval.

Heterogeneity was further explored by categorizing studies according to their diagnostic methods for GERD. The pooled effect size was not significant in Group A (objective diagnosis using pH monitoring or endoscopy; SMD = -0.16, 95% CI: -0.61 to 0.29) or Group B (symptomatic/questionnaire-based diagnosis; SMD = -0.10, 95% CI: -0.69 to 0.48). The between-group differences test (Q statistic) was not significant (p = 0.789), indicating that the diagnostic approach did not statistically explain the heterogeneity. Significant heterogeneity remained within each subgroup (Group A: I² = 71.47%, p = 0.007; Group B: I² = 84.99%, p < 0.001) (Figure 6).

**Figure 7:**
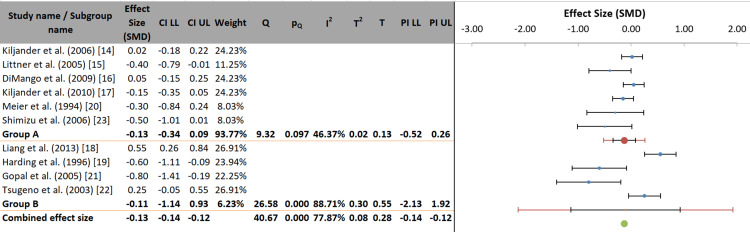
Forest plot of subgroup analysis by study design of the included studies. References correspond to Kiljander et al. [14], Littner et al. [15], DiMango et al. [16], Kiljander et al. [17], Liang et al. [18], Harding et al. [19], Meier et al. [20], Gopal et al. [21], Tsugeno et al. [22], and Shimizu et al. [23]. SMD: standardized mean difference; CI: confidence interval; LL: lower limit; UL: upper limit; PI: prediction interval.

Group A (RCTs) produced a non-significant pooled effect size (SMD = -0.13, 95% CI: -0.34 to 0.09), showing no apparent overall benefit of anti-reflux therapy on asthma exacerbations in randomized trials. Similarly, Group B (observational studies) had a non-significant pooled effect (SMD = -0.11, 95% CI: -1.14 to 0.93), but this estimate was imprecise due to very high heterogeneity (I² = 88.71%) (Figure 7).

When stratifying studies by GERD symptom status in the study populations, a primary source of overall heterogeneity was identified. The difference between subgroups was statistically significant (p = 0.001), explaining 56.26% of the variance in effects (pseudo R²). In symptomatic GERD populations (Group A), anti-reflux therapy had a statistically significant moderate effect (SMD = -0.41, 95% CI: -0.72 to -0.10), reducing asthma exacerbations. In asymptomatic or minimally symptomatic populations (Group B), no significant benefit was observed (SMD = 0.14, 95% CI: -0.21 to 0.49).

**Figure 8:**
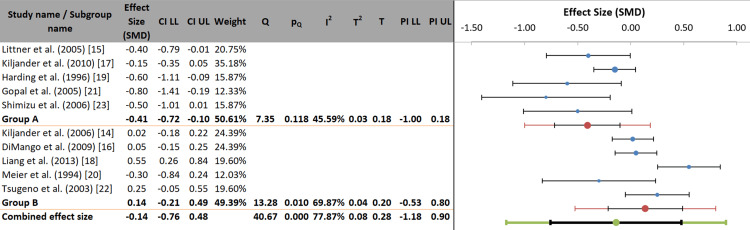
Forest plot of subgroup analysis by population GERD symptom status. References correspond to Kiljander et al. [14], Littner et al. [15], DiMango et al. [16], Kiljander et al. [17], Liang et al. [18], Harding et al. [19], Meier et al. [20], Gopal et al. [21], Tsugeno et al. [22], and Shimizu et al. [23]. GERD: gastroesophageal reflux disease; SMD: standardized mean difference; CI: confidence interval; LL: lower limit; UL: upper limit; PI: prediction interval.

This finding provides a significant clinical insight: the effect of GERD on asthma exacerbations appears to be modified by PPI treatment in patients with reflux symptoms, whereas treatment of silent reflux does not seem to influence asthma outcomes (Figure 8).

Despite subgroup stratification, substantial residual heterogeneity persisted within several subgroups (I² range: 58.3% to 88.7%). Clinical sources of residual heterogeneity included: (1) variability in asthma exacerbation definitions across studies, ranging from investigator-defined worsening to standardized criteria requiring systemic corticosteroids; (2) differences in baseline asthma severity and controller therapy; (3) varying PPI doses and treatment durations in RCTs (4-26 weeks); and (4) heterogeneity in GERD diagnostic methods even within subgroups. Methodological sources included: (1) variability in outcome measurement and reporting; (2) differences in follow-up duration; and (3) potential confounding in observational studies. These factors likely contributed to the observed between-study variance beyond the examined subgroup characteristics.

Discussion

The results of the systematic review and meta-analysis provide a broad overview of the multifaceted relationship between GERD and asthma exacerbations, explaining the situations in which an association occurs and vice versa. The combined analysis of all 10 studies also showed no statistically significant overall effect, consistent with the inconsistent results reported in the literature [3,7,14-16]. The importance of substantial heterogeneity (I^2^ = 77.87%), which our prespecified subgroup analyses sought to dispel, underscores the critical importance of this null finding. Such a high level of heterogeneity indicates that there is no one, universal (uniform) effect of GERD on asthma exacerbations, but rather that other important patient and study factors shape the relationship [25].

The most enlightening result was the subgroup analysis by population GERD symptom status. We have shown a significant divergence between our findings, where statistically significant, moderate improvements in asthma exacerbations were observed with anti-reflux therapy in populations that recruited patients with symptomatic GERD (SMD = -0.41), but not in those with asymptomatic or minimally symptomatic GERD (SMD = 0.14), and that the difference between subgroups was significant (p = 0.001). This essential difference resolves a paradox in the literature and aligns with the concept of asthma endotypes, in which disease-specific clinical phenotypes respond variably to targeted therapies [26].

The current study results confirm those of the groundbreaking trial by the American Lung Association Asthma Clinical Research Centers (2009), which found no benefit of esomeprazole in a wide-ranging population of asthmatics with poorly controlled disease, most of whom have silent reflux [7]. On the contrary, our finding supports the positive results of the study by Littner et al. (2005), which demonstrated that symptomatic acid reflux was necessary and showed a significant reduction in exacerbations with lansoprazole [15]. This implies that the symptomatic manifestation of GERD can be used to define a phenotype of reflux-initiated asthma that is more responsive to acid suppression. Still, objective but silent reflux will respond to no treatment.

This is in line with pathophysiological models in which symptomatic reflux may be associated with more proximal reflux or possibly increased esophageal sensitivity, which enhances vagally mediated bronchoconstriction [4]. The outcome also provides a possible explanation of the unfavorable results in DiMango et al. (2009), who specifically excluded symptomatic patients and targeted those who exhibited pathological but silent reflux, and showed no benefit of treatment [16]. This highlights the notion that the presence of abnormal esophageal acid exposure (pathophysiological GERD) is not sufficient to predict therapeutic outcome; rather, the transformation of this exposure into symptoms seems to be the most important factor.

There are significant clinical and health economic implications associated with this distinction. It presents a very strong argument against the objective use of PPI therapy in all asthmatics with objective evidence of GERD, which has been shown to have dismal results and has potential risks, including analytical susceptibility to respiratory infections (e.g., community-acquired pneumonia), *Clostridioides difficile* infection, and micronutrient deficiencies such as hypomagnesemia and vitamin B12 deficiency [27]. Rather, it promotes a targeted medical approach, which identifies a cure among patients who complain of concomitant reflux symptoms.

This approach is more likely to be effective and cost-effective, as it does not require unnecessary medication in non-responders and minimizes the burden of potential adverse effects [28]. This, in clinical terms, again boosts the external validity of an extensive history that dwells upon classic GERD symptoms (heartburn, regurgitation) as a critical element in the management of hard-to-treat asthma, as advocated by existing asthma management guidelines, which recommend that GERD treatment should be considered in people who are symptomatic first and foremost [29].

The study design subgroup analyses were not significantly different, despite their value. Equally, the diagnostic method (objective vs. symptomatic) proved to be less explanatory than the real symptom status of the population, which suggests that the definition of GERD to be included in the study is not as important as what patients (symptomatic or not) are being examined. This finding undermines the belief that more objective diagnostic traits (such as pH monitoring) are more appropriate for selecting treatment candidates in this situation; symptom burden appears to be the more clinically relevant prognostic indicator of treatment response. This is consistent with other extra-esophageal reflux manifestations, in which the degree of symptom correlation can frequently be the most significant factor in predicting treatment success [30].

These findings allow several mechanistic hypotheses to be formulated to inform future research. To begin with, symptomatic GERD can be an indicator of more serious or proximal refluxate, which increases the risk of microaspiration and direct airway irritation, triggering neutrophilic inflammation and bronchoconstriction [31]. Second, esophageal hypersensitivity, which is inextricably coupled with symptom perception in GERD and functional esophageal disorders, might also represent an augmented esophago-bronchial neural reflex arc. The increased neural crosstalk may imply that even small volumes of reflux in a sensitive esophagus may cause considerable bronchospasm, which acid suppression would counter. This neural hypersensitivity may not be present in patients with silent reflux, or acid suppression may be less effective at controlling bronchial tone [32]. Third, the symptoms might be more closely associated with nocturnal reflux, which is a single cause of asthma symptoms and exacerbations, due to the extended recumbent posture and elevated vagal tone [33].

The publication bias assessment, which should be viewed with caution, indicates that smaller studies with null or negative outcomes may be underrepresented in the literature, potentially leading to an exaggeration of perceived efficacy in selected subgroups [12]. Such asymmetry may also be indicative of actual heterogeneity, in which smaller, earlier studies showing large effects in highly selected, symptomatic populations were published. In contrast, larger, more pragmatic studies in broader, less selected populations yielded null outcomes [34].

This meta-analysis redefines the study problem by showing that the interaction varies by patient phenotype, with symptomatic GERD serving as a significant effect modifier. The data do not confirm the existence of a universal relationship, but instead, symptomatic GERD is recognized as a manageable phenomenon in the heterogeneous asthma group. It also guides the design of future studies, which must aim to validate biomarkers (e.g., pepsin in sputum and impedance-pH patterns) that can better identify the reflux-responsive asthmatic in the absence of symptom report, and on interventions other than acid suppression (e.g., reflux inhibitors or neuromodulators) to patients with refractory symptoms [35].

The substantial residual heterogeneity observed even after subgroup stratification warrants careful consideration. The variability likely reflects the inherent complexity of the GERD-asthma relationship, in which multiple interacting factors, including reflux characteristics (acidic vs. non-acidic, proximal vs. distal), individual patient susceptibility (esophageal sensitivity, airway hyperresponsiveness), and treatment factors (PPI dose, adherence, duration), collectively influence outcomes. The persistence of heterogeneity suggests that our subgroup analyses, while informative, could not fully capture all relevant effect modifiers. Future studies should employ standardized exacerbation definitions and more refined phenotyping of both GERD and asthma to reduce heterogeneity.

Limitations

Several important limitations must be acknowledged. First, pooling distinct study designs (RCTs evaluating interventions and observational studies evaluating exposures) is methodologically problematic, as they address fundamentally different clinical questions. Although separate primary analyses by study design were conducted, the exploratory combined analysis should be interpreted with caution, and it was recognized that a single pooled estimate across designs might be clinically misleading. Second, converting diverse outcome measures, including event counts, IRRs, odds ratios, and continuous scores, into a common SMD metric may introduce inaccuracies. While we followed established conversion methods (Hasselblad, Cox transformations), these approaches rely on assumptions (e.g., normal distribution of underlying liability) that might not hold perfectly across all studies. Sensitivity analyses limited to studies reporting directly compatible outcomes yielded similar results, partially mitigating this concern. Third, most studies focused on acid-suppressive PPI therapy, limiting generalizability to other anti-reflux interventions. Finally, restriction to English-language publications may have introduced language bias.

Future directions

Future studies should focus on pragmatic RCTs that stratify asthmatic patients by GERD symptom status to test the hypotheses generated here. Uniform, clinically meaningful definitions of asthma exacerbations (e.g., requiring systemic corticosteroids or hospitalization) are needed to improve comparability. Mechanistic studies should investigate the link between symptomatic reflux and airway hyper-responsiveness, including esophageal hypersensitivity and neural reflex pathways. Cost-effectiveness studies comparing symptom-driven GERD therapy versus empirical treatment are warranted. Finally, the utility of advanced diagnostics, such as impedance-pH monitoring, for identifying reflux-responsive asthma should be further explored.

## Conclusions

This systematic review and meta-analysis did not identify a significant overall effect of GERD or its therapy on asthma exacerbations across all populations. However, a clinically important and statistically significant moderating factor emerged: anti-reflux therapy appeared to have a protective effect specifically in asthmatic patients with symptomatic GERD, but not in those with silent reflux. This finding should be considered hypothesis-generating rather than definitive, given the substantial heterogeneity, methodological limitations of pooling diverse study designs, and the small number of studies available for subgroup analysis. The results suggested that symptomatic GERD might represent a treatable trait within the heterogeneous asthma population, but this requires confirmation through prospective RCTs that stratify patients by symptom status a priori. Pending such confirmatory evidence, a pragmatic approach would prioritize the identification and treatment of GERD symptoms in asthmatics rather than empirical therapy for all patients with objective evidence of reflux. This symptom-oriented strategy aligns with current guideline recommendations while acknowledging the need for higher-quality evidence to establish the role of anti-reflux therapy in asthma management definitively.
